# The Fork in the Road: Histone Partitioning During DNA Replication

**DOI:** 10.3390/genes6020353

**Published:** 2015-06-23

**Authors:** Anthony T. Annunziato

**Affiliations:** Biology Department, Boston College, 140 Commonwealth Avenue, Chestnut Hill, MA 02467, USA; E-Mail: anthony.annunziato@bc.edu; Tel.: +617-552-3812; Fax: +617-552-2011

**Keywords:** chromatin, nucleosome, tetramer, histone, acetylation, methylation, replication, splitting, epigenetics

## Abstract

In the following discussion the distribution of histones at the replication fork is examined, with specific attention paid to the question of H3/H4 tetramer "splitting." After a presentation of early experiments surrounding this topic, more recent contributions are detailed. The implications of these findings with respect to the transmission of histone modifications and epigenetic models are also addressed.

## 1. Introduction

"When you come to a fork in the road, take it!"

Y. Berra

Much of the focus of chromatin research in the past 40 years has been aimed at uncovering what happens to nucleosomes during transcription and DNA replication. The earliest electron micrographs of nucleosomal fibers *in situ* presented two opposite extremes of chromatin function: either tandemly arrayed ribosomal genes crowded with RNA polymerases, packed shoulder-to-shoulder [1]; or transcriptionally inert chromatin fibers, often prepared from avian erythrocytes [2,3,4]. In neither case could the dynamic behavior of histones be discerned. Even high resolution electron micrographs of chromatin replication initially revealed little concerning the details of histone-DNA interactions, except to establish that nucleosome assembly occurred remarkably rapidly on new DNA [5,6].

With respect to chromatin replication, questions surrounding the distribution of parental histones at the fork, *de novo* nucleosome assembly, and the ability of histone posttranslational modifications (PTMs) to serve as agents of epigenetic inheritance are intimately linked. Relationships among these interwoven processes are still being worked out, and, not surprisingly, have been the subjects of numerous review articles [7,8,9,10,11,12,13,14]. What follows is an examination of our current understanding of the manner in which parental histones (especially H3 and H4) are segregated during DNA synthesis. While the older history of this topic will be touched upon, more recent findings will be described in some detail. Related implications for the epigenetic inheritance of histone PTMs will also be examined.

## 2. Taking the Fork in the Road

Histone proteins persist for many generations in both proliferating and nonproliferating cells [15,16]. Moreover, experiments in which DNA replication was allowed to occur in the absence of concurrent protein synthesis (for example, in the presence of cycloheximide or in isolated nuclei) revealed that parental histones were transferred to nascent chromatin almost instantaneously [17,18,19,20,21,22]. Although there had been initial speculation that pre-existing histones might preferentially sort with the leading nascent strand, it is now generally accepted that old histones are partitioned to both new daughter DNA helices as the fork progresses, in clusters that range on the order of approximately 7-10 nucleosomes (reviewed in [8]). Several (thus far) unanswered questions follow from this model. First, the rules that may control parental histone segregation (assuming that the process is not completely stochastic) are unknown. It would be of considerable interest to know whether a particular locus follows the same pattern every cell cycle, and if/how the limited cooperativity of histone distribution is regulated. In addition, discussions of the epigenetic inheritance of PTMs must take into account that only one of the replicated copies of a particular DNA sequence can receive parental histones, and that this asymmetry might extend over a considerable distance. Also to be considered are the degree to which nucleosomes are routinely well "positioned," and the extent to which such positioning is disrupted following DNA replication [23]. These topics will be discussed again in a subsequent section.

## 3. The Stability of the H3_2_H4_2_ Tetramer and Tetramer "Splitting"

### 3.1. Early Studies

Investigations of histone segregation and nucleosome assembly inevitably encompass considerations of the long-term stability of histone complexes and the mixing of new and old H3/H4 dimers in nascent nucleosomes. Before describing recent approaches to this problem, it will be helpful to briefly review earlier examinations of these topics.

Initial studies of H3/H4 tetramer stability involved either following the fate of tagged histones over the course of multiple cell cycles or electron microscopy. In the case of the former, the tags typically included fluorescent adducts or "dense" amino acids containing heavy atomic isotopes. Prior *et al.* examined the persistence of H3-H3 interactions in the slime mold *Physarum* using the fluorescent reagent iodoacetoxypyrene, which can be linked to cysteine residues [24]. In the case of H3, this is the sole cysteine, which occurs at position 110. Notably, C-110 lies at the H3-H3 interface in the H3/H4 tetramer, and in fact, two H3/H4 dimers can be covalently linked *in vitro* by disulfide binds [25]. *Physarum* was used in the experiments of Prior *et al.* because of its ability to absorb histones placed onto its surface and assemble them directly into nucleosomes [24,26].

Monomeric pyrene emits blue light, while two pyrene molecules in close proximity fluoresce green. Thus, the emission spectra of H3/H4 dimers containing of pyrene-labeled H3 (AP-H3) can be used to monitor tetramer stability *in vivo*. Shortly after being placed onto the *Physarum* plasmodium, AP-H3 appeared blue in the cytoplasm and in nuclei, as expected for free H3 or H3/H4 dimers. Blue fluorescence then gradually diminished to be replaced by green, indicating AP-H3 incorporation into nucleosomes (which was verified). Green fluorescence subsequently persisted for at least 90 hours, while blue fluorescence did not return. It was therefore concluded that, once assembled, the great majority of H3/H4 dimers remain intact over several generations. Further evidence for the long-term stability of (most) H3/H4 tetramers came from pulse-chase experiments, in which *Physarum* was sequentially exposed to AP-H3 followed by unlabeled H3. Even after a 36 hr "chase," there was no evidence for green excimer depletion or blue fluorescence recurrence in isolated nucleosomes, fully consistent with the preservation of H3/H4 tetramer integrity through several rounds of DNA replication [24].

The use of heavy atomic isotopes to track macromolecules during DNA replication is, of course, a long-established methodology [27]. With respect to nascent chromatin, histones labeled *in vivo* with dense amino acids (containing ^13^C and ^15^N) have been used to examine the manner in which the histone octamer is assembled. In these experiments, complexes containing new or old histones are chemically crosslinked and resolved from each other by equilibrium density centrifugation. While some discrepancies in the literature surrounding this topic persist, especially regarding the deposition of new H2A/H2B dimers, the consensus has emerged that new H3/H4 tetramers are conservatively assembled entirely of new histones (reviewed in [8]). By modifying experimental protocols to include an extended "chase" in the presence of normal (light) amino acids, the stability of histone complexes over multiple cell generations has also been monitored. Within the parameters of resolution afforded by density gradient centrifugation, such studies have yielded results supporting the conservative transfer of intact H3/H4 tetramers to new DNA [28,29,30,31], consistent with the experiments of Prior *et al.* [24]. Whether the H3/H4 tetramer is temporarily disrupted into heterotypic dimers was not determined. 

As noted above, electron microscopy has also been used to inquire how pre-existing histones are partitioned at the replication fork. To exclusively examine the transfer of old histones to new DNA, experiments have typically been performed using cell-free systems, such as SV40 viral minichromosomes replicated *in vitro*. Under these conditions, there are no new histones available for *de novo* nucleosome assembly. Whether by direct staining of chromatin fibers or through psoralen cross-linking, no "half-nucleosomes" have been observed following chromatin replication *in vitro* [20,32,33]. Evidence against parental octamer or tetramer splitting has also been obtained through the use of micrococcal nuclease or supercoiling assays as probes of chromatin replicated in the absence of histone synthesis [17,18,21,22,34,35]. In addition, experiments analyzing the replication of SV40 minichromosomes reconstituted with chemically cross-linked octamers have shown that nucleosomes need not be disrupted for replication to proceed or for old octamers to be transferred to new DNA [36]. It of course remains formally possible that by preventing *de novo* nucleosome assembly (and perhaps histone chaperone function), these experimental protocols interfere with the normal process of histone distribution at replication forks. Nevertheless, the cumulative results from many laboratories over many years have been consistent with either the preserved integrity or the rapid reassembly of parental H3/H4 tetramers during DNA synthesis. More recent approaches to the question of parental histone partitioning will be discussed in the following section.

### 3.2. Splitting Revisited

Central to the study of histone segregation and tetramer splitting is the ability to distinguish new from old histones. One method of accomplishing this is differential epitope-tagging, in which two or more copies of the same histone are selectively tagged and expressed at different times in the same cell. This approach was taken by Katan-Khaykovich and Struhl, who used budding yeast as a model system [37]. In this organism, the sole H3 variant is homologous to mammalian H3.3, and is deposited in both replication-dependent and replication-independent manners [38,39,40]. In the experiments of Katan-Khaykovich and Struhl, H3 was tagged with either the VSV glycoprotein (VSVG), and expressed under control of a methionine-repressible *MET3* promoter; or with HA, and regulated by the *GAL1* promoter. The yeast strain used lacked the endogenous H3 genes, and it was confirmed that each tagged H3 could individually support cell viability. To test for H3/H4 tetramer splitting, cells were first grown in medium containing raffinose and lacking methionine to induce VSVG-H3 (to represent "old" H3). Cells were then shifted to medium containing methionine (to repress VSVG-H3 expression) and, after a chase period, were grown in galactose to induce HA-H3 (representing "new" H3). Mononucleosomes were then subjected to sequential immunoprecipitation, and the occupancy of VSVG-H3 and HA-H3 was analyzed by PCR at a total 14 different loci. It was found that co-occupancy of new and old H3 was generally low, indicating an absence of splitting and mixing, except at loci showing high levels of transcriptional activity and histone exchange. Control experiments verified that when simultaneously co-expressed, co-occupancy of both forms of H3 was high, as expected. As all loci must be replicated once per cell cycle, these results indicate that tetramer splitting does not normally occur during DNA replication, but rather is a consequence of histone exchange during dynamic transcription, as is likely mediated by chromatin remodeling complexes and soluble histone-bearing chaperones (see section 4, below).

The use of stable atomic isotopes and density gradient centrifugation to distinguish between new and old histone complexes was discussed in a previous section. More recent studies using dense isotopes (typically referred to as SILAC, *i.e.*, Stable Isotope Labeling of Amino Acids in Cell Culture) have employed mass spectrometry to quantify the mixing of new and old histones. Xu *et al.* used SILAC and epitope-tagging to examine tetramer splitting in HeLa cells [41]. In separate experiments, either FLAG-H3.1 or FLAG-H3.3 was induced in the presence of normal-density lysine to represent old histone. Flag-H3 expression was then turned off for a two-day chase period. Cells were subsequently synchronized, and grown in medium supplemented with dense lysine (containing ^13^C and ^15^N) for up to 72 hours to label "new" histones. Mononucleosomes were then prepared and affinity-purified using anti-Flag antibodies. Individual co-purified histones were then electrophoretically separated, excised from gels, and analyzed by tandem mass spectrometry (MS/MS). It was found that Flag-H3.1 remained associated with normal-density H3.1 and normal H4, as expected, had the old histone tetramers remain intact during multiple rounds of DNA replication. Notably, in these experiments, H2A and H2B histones co-purified with old Flag-H3.1 were ~50% heavy, demonstrating H2A/H2B exchange is independent of tetramer dissolution.

When the experiments were modified to follow Flag-H3.3, a different story emerged. After two cell cycles in dense medium, ~23% of the native H3.3 that co-purified with Flag-H3.3 was density-labeled, indicating significant H3.3/H4 tetramer splitting. Notably, when Flag-H3.3 cells were cultured with dense lysine for 72 hours in the presence of inhibitors of DNA replication, H3.3/H4 tetramer splitting was significantly reduced—in the case of aphidicolin, by ~75% relative to untreated controls. Under the same conditions the deposition of H3.1 was effectively eliminated. The authors concluded that most replication-*coupled* assembly of H3.3_2_H4_2_ tetramers involves the mixing of new and old H3/H4 dimers, and that replication-*independent* deposition of H3.3 is accomplished by the cooperative assembly (and not splitting) of two new H3.3/H4 dimers [41]. This is in contrast to the conclusions of Katan-Khaykovich and Struhl, cited above [37].

A more recent examination of H3.3_2_H4_2_ tetramer splitting using the differential epitope-tagging method in HeLa cells has been presented by Huang *et al.* [42]. In this study, two separately inducible tagged forms of H3.3 (either Flag- or HA-) were placed into the same stable cell line. Induction protocols were chosen to serially express either HA-tagged H3.3 ("old") or Flag-H3.3 ("new"). Mononucleosomes were then prepared and subjected to a single immunoprecipitation with either anti-Flag or anti-HA antibodies (to create separate pools of new or old nucleosomes) or purified by sequential chromatin immunoprecipitation (ChIP) using both antibodies to selectively harvest "split" mononucleosomes. Immunoprecipitated mononucleosomal DNA was analyzed by sequencing (ChIP-Seq). To specifically examine the turnover rate of H3.3 in nucleosomes, the induction protocol was altered. HA-H3.3 expression was induced for 48 hours and then switched off. Following chase periods of 0 h, 24 h, and 48 h, mononucleosomes were analyzed by ChIP-Seq with anti-HA antibodies. All results were then compared to a genomic RNA-Seq analysis. By quantitating H3.3 occupancy, H3.3 turnover, and H3.3_2_H4_2_ tetramer splitting relative to the RNA-Seq data, the authors drew the following conclusions: (1) H3.3 localization and exchange (turnover) occurs at transcriptionally active regions, in agreement with previous findings [43,44,45,46,47,48]; (2) H3.3 turnover at Transcription Start Sites (TSS) follows a bimodal distribution (either "yes" or "no") instead of being linearly correlated with the level of transcription; (3) H3.3_2_H4_2_ tetramer splitting is enriched at active genes, and is not strictly correlated with H3.3 turnover (*i.e.*, not all turnover involves splitting); and (4) H3.3 nucleosomes with the highest level of tetramer splitting are located at cell-type specific enhancer regions [42].

A potential complication with the experimental approach used by Huang *et al.* is that, unlike the case with yeast (in which the cellular H3 genes were deleted), the endogenous HeLa H3.3 genes remained present and transcriptionally active. As the epitope-tagged H3.3 histones represented only 5–10% of total H3.3 histones, most tetramer splitting will, of course, involve the deposition of native H3.3. Because the split tetramers were captured and scored by sequential ChIP using two different antibodies, it is clear that the vast majority of splitting events remained undetected. A related problem is that nucleosomes with the highest splitting rates may exchange out *both* H3/H4 dimers over time and be lost to ChIP analysis entirely. The authors addressed these issues statistically, based on the relative abundance of tagged H3.3, and by compensating for the H3.3 "turnover index." Yet the possibility remains that the true extent of splitting may differ from the estimated levels, especially as not all H3/H4 exchange involves tetramer disruption (according to the same authors) [42].

Still to be explained are the discrepancies between the findings of Xu *et al.* [41] and Katan-Khaykovich and Struhl [37] in regard to H3.3_2_/H4_2_ tetramer splitting as a function of DNA replication. On one hand, they may reflect differences between the fungal and mammalian cell systems used, although in light of the fact that budding yeast H3 is homologous to H3.3, one might have predicted H3/H4 tetramer splitting to be more pervasive in yeast, and perhaps driven in part by DNA replication as well as transcription. However, this is not the case [37]. In contrast, the splitting of H3.3 nucleosomes in HeLa cells appeared to be largely reliant on DNA replication, and thus was reduced in the presence of replication inhibitors [41]. Several observations may be made here. First, in the experiments of Xu *et al.*, replication was inhibited for up to 72 hours. Such lengthy treatments may trigger cell-cycle checkpoints and significantly decrease RNA synthesis as well as DNA replication. Second, it should be noted that, in the same experiment, the exchange of H2A/H2B dimers was reduced by as much as 60%. As H2A/H2B exchange has long been linked to transcriptional activity (reviewed previously in [8,49]), its decrease may also reflect a drop in transcription-coupled nucleosome dissolution and tetramer splitting. Moreover, although H3.3 is deposited both in and out of S phase [45,47], there is evidence that in mammalian cells H3.3 is not deposited at replication forks under normal conditions [47]. This would seem to preclude a model calling for a major role for DNA synthesis in H3.3_2_H4_2_ tetramer splitting. It therefore seems possible that the decrease in tetramer splitting observed by Xu *et al.* may have been due to the repression of transcription, not DNA synthesis *per se*. Another factor to be considered is that the regulation of H3/H4 exchange can be cell-type specific [50,51].

## 4. The Involvement of Histone Chaperones

Free H3/H4 tetramers are stable under conditions of physiological ionic strength and pH [52]. It was therefore not expected that nucleosome assembly complexes and histone chaperones should carry H3/H4 dimers, not tetramers [53,54,55,56] (reviewed in [7,8,11,12,13,57]). With respect to H3/H4 tetramer splitting, related questions that follow concern the association of chromatin assembly factors with the H3/H4 dimer, and the processing of histones at the replication fork.

The chaperone Asf1 binds an H3/H4 dimer through its association with the C-terminus of histone H3, thereby preventing the assembly of an H3_2_H4_2_ tetramer [58,59,60,61,62]. Asf1 also interacts with the p60 subunit of CAF-1 [63,64,65] and is a member of several chromatin assembly complexes [53,66,67,68,69]. Importantly, Asf1 can be localized to replication forks [70,71] and has been found to associate with MCM helicases [72,73]. Moreover, the helicase subunit MCM2 itself binds histone H3 [74,75,76,77]. Crystallographic analysis of the association of H3 and H4 with recombinant fragments of human MCM2 have described an (H3/H4)_2_MCM2_2_ hexamer [77]. This complex was also observed in solution studies performed at 0.5 M NaCl; one of the MCM2 proteins dissociates at 1.5 M NaCl [77]. When the 1-156 region of human Asf1a was included in the assembly solution, a quaternary complex comprising Asf1-H3-H4-MCM2 in a 1:1:1:1 ratio was formed [77]. The same complex was also identified in GST pull-down studies [78]. As only one MCM2 protein is in the replisome, this may be the physiologically relevant complex. 

The presence of Asf1, which has H3/H4 tetramer splitting ability, at the sites of DNA unwinding during replication clearly raises the possibility that this chaperone mediates the passing of parental H3/H4 to new DNA through transient tetramer disruption (see [72,73,77]). However, it must be stressed that the actual processes by which histones are transferred from old DNA to new are unknown. It is possible that the CAF-1 complex (which can be targeted to the replisome through its association with PCNA [79,80]) is involved, especially as CAF-1 itself is associated with Asf1a and Asf1b *in vivo* [53]. Of course, CAF-1 is also responsible for the deposition of newly synthesized H3/H4, and thus its capacity for capturing parental H3/H4 may be limited [8]. As with Asf1, isolated H3/H4 predeposition complexes have been shown to carry dimers [53,55], yet there is also evidence that yeast CAF-1 can bind an H3/H4 tetramer, both *in vitro* [81] and *in vivo* [82], and that deleting the CAF-1 subunit *CAC1* reduces histone turnover at the 5' ends of genes in yeast [83]. In this regard, it would be of considerable interest to know whether CAF-1 ever binds pre-existing histones during chromatin replication. This might be accomplished through the tagging and labeling techniques discussed above, and through controlled histone expression in synchronized cells. Importantly, it has been demonstrated that the FACT chromatin remodeling complex is associated with the replisome and is able to partner with parental H3 during DNA replication ("old" H3 was identified by the absence of acetyl-K56, a nascent H3 mark) [76]. Thus, while there is evidence for the association of nuclear Asf1 and FACT with parental H3/H4 [72,73,76], in the absence of other evidence, it must be assumed that cooperation between Asf1 and CAF-1 is restricted to the deposition of newly synthesized histones.

## 5. Epigenetic Considerations

### 5.1. An Epigenetic Code?

Much of the interest surrounding histone segregation has been focused on its potential to serve as a mechanism for the epigenetic inheritance of posttranslational modifications. Although routine tetramer splitting during replication can now be excluded (with the possible exception of some H3.3 nucleosomes), the manner in which histone modifications are reestablished after chromatin assembly remains the subject of considerable research effort, and has been comprehensively reviewed in recent years [9,11,12,84,85,86,87,88]. An in-depth discussion of epigenetic inheritance will therefore not be attempted here. Instead, a brief overview of the topic will be presented, concluding with a discussion of more recent contributions concerning the preservation of histone modifications during DNA replication. 

The demonstration of histone PTMs on mitotic chromosomes argues strongly for the cross-generational transfer of these epigenetic signals [89,90]. Moreover, the underacetylation of the inactive X chromosome in females can be taken as evidence that these marks (or their removal) are regulatory in nature [89,90,91]. The maintenance of histone methylation during mitosis is another example of a likely epigenetic mechanism [92,93,94,95,96,97]. If histone modifications are to pass from one generation to the next, then their persistence during DNA replication must be unimpeded and, at the very least, not overwritten. This question was addressed in an early ChIP analysis of parental nucleosomes using antibodies that recognize acetylated H4. Cells were labeled for one generation with [^14^C]thymidine to uniformly label "bulk" chromatin DNA. Newly replicated DNA was then labeled *in vivo* with [^3^H]thymidine in the presence of cycloheximide (to eliminate the deposition of new histones) or with [^3^H]TTP in isolated nuclei during run-on replication [98]. In both cases it was found that the percentage of "new" nucleosomes containing parental acetylated-H4 was equal to the percentage of total nucleosomes with acetylated H4 [98]. This was in sharp contrast to nucleosomes assembled *de novo* with newly synthesized histones (*i.e.*, in the absence of cycloheximide), which were highly enriched in the immunopellet due to the acetylation of new H4 [99]. Two conclusions follow from these observations: 1) chromatin is not obligatorily acetylated to allow for replication, and thus differences between active and inactive chromatin are potentially preserved; and 2) acetylation is not always *erased* during replication, raising the possibility of an epigenetic marking. These findings were later expanded to include other PTMs on segregated histones [55].

### 5.2. Histone Methylation

Epigenetic transfer of the repressed chromatin state through inherited histone methylation (and/or histone-binding proteins) has received considerable attention. (In the interest of conciseness, RNAi-mediated mechanisms will not be discussed herein; for reviews see [100,101,102,103,104]). One repressive factor that has been examined is the evolutionarily conserved Polycomb complex PRC1, which binds H3K27me3 [105,106]. In an early report, it was demonstrated that PRC1 remains bound to DNA during the replication of SV40 minichromosomes *in vitro*, in a system utilizing a HeLa cell S100 extract [107]. Subsequent studies using a T7 bacteriophage system have shown that eukaryotic proteins are not required to retain PRC1 during DNA synthesis [108]. This latter experiment raises the possibility that the retention of PRC1 can be directed by DNA alone. Of course, *in vivo* DNA replication would occur in the context of chromatin possessing Polycomb complexes and H3K27me3. A model for Polycomb retention has been proposed that incorporates the ability of the PRC1 subunit PSC to self-associate while dynamically bound to nucleosomes, thereby providing a bridge to traverse the advancing replisome [109]. This could be facilitated through the association of the PCR2 methyltransferase with pre-existing trimethylated H3, thereby causing allosteric activation of its enzymatic activity [110]. In this manner, histone H3 in nearest neighboring (new) nucleosomes would be methylated, permitting the transmission of the H3K27me3 mark [110,111].

The persistence of histone methylation following DNA replication has been a matter of some controversy. Mazo and colleagues, working with *Drosophila* embryos, reported that the Polycomb proteins Pc and E(z) persisted on newly replicated DNA, but that all H3K4me3 and H3K7me3 marks were lost, and did not significantly reappear for at least an hour, *i.e.*, until cells had exited S phase [112]. The authors proposed that parental histones are dissociated during replication and demethylated before replacement, while Polycomb-complex proteins and histone-modifying enzymes are retained [112,113]. In contrast, diametrically opposite observations were made by Strome and colleagues, who examined the transmission of the PRC2 complex and H3K27 methylation in the nematode worm *C. elegans* [114]. Two experimental designs were tested. In one case, it was found that H3K27me3 marks on sperm chromatin were retained after multiple rounds of replication during early development, even after fertilization of oocytes that lacked the maternal PRC2 methyltransferase; however, H3 in the maternally derived chromosomes remained unmethylated, as expected [114]. In the reciprocal experiment, unmethylated H3 in sperm from worms lacking PRC2 did not become methylated following fertilization of oocytes possessing PRC2 activity, although maternally derived chromosomes kept the methyl mark. Notably, in the adult germ line, all chromosomes eventually became methylated through the enzymatic activity of maternal PRC2 [114]. The difference between flies and worms concerning the heritability of histone methylation has not been resolved, especially as both sets of experiments rely on embryonic systems. It may be that in flies interactions between methylating enzymes and DNA sequences regulate the transmission of repressive chromatin PTMs, while in worms the PTMs themselves provide the requisite cellular memory.

With respect to histone PTMs in mammalian cells, a somewhat complex mechanism of epigenesis seems to be operative. Through affinity purification of newly replicated chromatin labeled with bio-dUTP in the presence of cycloheximide (to prevent new histone synthesis), it was found that parental H3K9 and H3K27 methylation marks could be passed to replicated nucleosomes [115]. In addition, several studies have described the gradual reestablishment of H3 methylation marks over the course of the cell cycle (or cycles) following S phase [9,116,117,118,119]. In an experimental system involving the transient recruitment of HP1-alpha and H3K9-specific methylases to a reporter gene in mouse embryonic fibroblasts, it was found that the H3K9me3 mark was stably maintained for several generations, even in the absence of continued methylase activity [120]. However, inducing transcription of the reporter under these conditions resulted in the gradual loss of histone methylation, indicating that the repressive mark is dynamic in nature [120]. By combining the bio-dUTP chromatin capture method with pulsed density-labeling of histones (SILAC), Alabert *et al.* have examined the reestablishment of histone PTMs on new and old histones immediately after DNA replication and over the course of the subsequent cell cycles [121]. Importantly, no marks were underrepresented on old histones in newly replicated chromatin, relative to total chromatin. This is in agreement with the early ChIP results cited above, concerning the potential transmittance of parental histone PTMs [55,98]. This would be in contrast to the complete erasure of PTMs, as observed in experiments using *Drosophila* embryos [112]. Based on their results, Alabert *et al.* proposed two different modes of PTM establishment following DNA replication: 1) retention of most parental PTMs on old histones, coupled with the generation of most "parental"-type PTMs on new histones within the same cell cycle; and 2) gradual establishment of the H3K9me3 and H3K27me3 marks on new H3, requiring more than one generation to complete [121]. As these marks are of course diluted in half following replication, a potential loss of cellular memory could be a problem. However, this is counterbalanced by the continual modification of both new and old histones, such that the total modification milieu is reestablished over time [121], as previously observed [9,116,117,118]. As proposed by Zhu and colleagues, the combined effect of these methyl marks on both new and old H3 could be sufficient to maintain regional silencing, as long as a certain critical threshold of H3 methylation is maintained [9,85].

### 5.3. Histone Acetylation

Histone acetylation is an integral aspect of chromatin replication and assembly, as evidenced by the long-established acetylation of newly synthesized H3 and H4 [73,122,123,124,125,126,127,128,129,130] (reviewed in [8,10,131,132]). What has received less attention is the transmission of parental histone acetylation during DNA synthesis. Scharf *et al.* have examined the kinetics of the equalization of acetylation between new and old H4 proteins following nucleosome assembly; however, the time frames chosen (hours) did not specifically address the preservation of acetylation on pre-existing histones immediately after replication [116]. Alabert *et al.* have closely examined the levels of H3/H4 acetylation in parental segregated nucleosomes, as compared to the levels in the genome as a whole [121]. In agreement with earlier findings (discussed above, [98]), no significant differences in the acetylation of histone H4 were observed, fully consistent with the preservation of acetylation following passage of the replication fork. An elevated acetylation of old H3 at K23 was observed (supplementary Fig. 1C, [121]), but not to the degree found for newly synthesized H3 [121].

The underlying hypothesis with respect to the epigenetic inheritance of histone PTMs is that new chromatin fibers will eventually acquire the same marks as parental chromatin, at the same level and frequency. Given that newly synthesized H4 and H3 histones carry site-specific acetylation patterns (reviewed in [8]), a critical aspect of epigenesis will necessarily entail timely histone deacetylation after chromatin assembly. In this regard, it has been found that new H3 and H4 are substantially deacetylated within 30–60 min of deposition (e.g., [122,123,124,133]). Deacetylation is an essential feature of the assembly process, as chromatin replicated in the presence of a deacetylase inhibitor fails to mature properly and remains highly sensitive to digestion by DNase I [134]. This may be due to the failure to acquire proper chromatin higher order structures [135,136]. How the requisite deacetylation is coordinated with the establishment of parental PTMs on new H3/H4 is a topic ripe for further investigation.

It can be argued that histone acetylation turnover is too extensive [137,138], or that the acetylation "code" is generally too inexact [139], for it to act as a major epigenetic signal. However, this may presuppose that "epigenetics" is operative at single nucleosome resolution. Thus, an important consideration is the degree to which nucleosomes become repositioned after DNA synthesis. In the budding yeast *S. cerevisiae* it has been found that nucleosome positioning is quickly reestablished after the replication of rDNA [140]. However, Rando and colleagues have found that parental H3 in yeast tends to accumulate at the 5'-ends of moderately active genes [83]. It was therefore proposed that limited spreading of parental histones occurs after replication, accompanied by H3 pass-back and exchange during transcription [83]. Eventually, precise nucleosome positioning is reestablished in yeast [23,141], evidence that the system can tolerate short-term disruption. 

## 6. Conclusions

The cumulative evidence over the past 40 years does not support H3/H4 tetramer splitting as a significant mechanism for either the partitioning of histones or the transmission of histone PTMs after DNA synthesis. Indeed, the finding that many H3/H4 tetramers are asymmetrically modified puts constraints on the splitting model, even in theory [142,143]. Nevertheless, there is ample experimental support for the preservation of histone modifications following replication, although in some instances it takes an extended period to fully reestablish the parental pattern. When taken together with the observations that multiple histone PTMs are often correlated with one another [144,145], and that histone positioning on new DNA may be inexact (at least initially), a more zonal model may be operative. As suggested by Zhu and colleagues [9,85], a "buffer" system may be able to tolerate dynamic methylation, acetylation, *etc.* within certain parameters, to maintain the distinction between transcriptionally active and repressed regions after chromatin replication and assembly. Whether or not this meets the criteria for an epigenetic mechanism may largely depend on one's definition of the term. 
